# An Update Regarding the Bioactive Compound of Cereal By-Products: Health Benefits and Potential Applications

**DOI:** 10.3390/nu14173470

**Published:** 2022-08-24

**Authors:** Anca Corina Fărcaș, Sonia Ancuța Socaci, Silvia Amalia Nemeș, Oana Lelia Pop, Teodora Emilia Coldea, Melinda Fogarasi, Elena Suzana Biriș-Dorhoi

**Affiliations:** 1 Department of Food Science, Faculty of Food Science and Technology, University of Agricultural Sciences and Veterinary Medicine Cluj-Napoca, Manastur 3-5, 400372 Cluj-Napoca, Romania; 2 Institute of Life Sciences, University of Agricultural Sciences and Veterinary Medicine Cluj-Napoca, Manastur 3-5, 400372 Cluj-Napoca, Romania; 3 Department of Food Engineering, Faculty of Food Science and Technology, University of Agricultural Sciences and Veterinary Medicine Cluj-Napoca, Manastur 3-5, 400372 Cluj-Napoca, Romania

**Keywords:** bioactive compounds, bioavailability, cereal by-products, health benefits, waste

## Abstract

Cereal processing generates around 12.9% of all food waste globally. Wheat bran, wheat germ, rice bran, rice germ, corn germ, corn bran, barley bran, and brewery spent grain are just a few examples of wastes that may be exploited to recover bioactive compounds. As a result, a long-term strategy for developing novel food products and ingredients is encouraged. High-value compounds like proteins, essential amino acids, essential fatty acids, ferulic acid, and other phenols, tocopherols, or β-glucans are found in cereal by-products. This review aims to provide a critical and comprehensive overview of current knowledge regarding the bioactive compounds recovered from cereal by-products, emphasizing their functional values and potential human health benefits.

## 1. Introduction

The European Union’s (EU) largest manufacturing sector is certainly the food and beverage industry [1]. Only biomass derived from food and feed crops, dedicated energy crops, trees, and agriculture residues now offers an accessible source for chemicals and products with the high added value among all known sustainable resources (solar, wind, geothermal) [2]. The main nutritionally exploitable wastes can be considered fruits and vegetables, meat and dairy, bakery, cereals and breweries, and aquaculture by-products [3,4].

**Figure 1:**
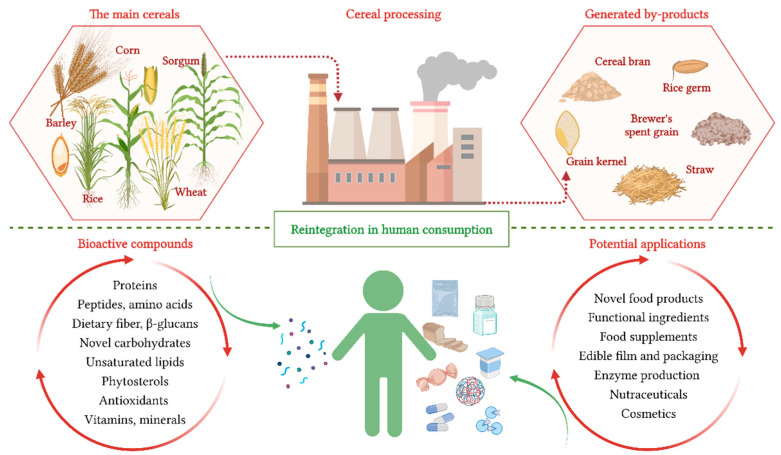
Reintegration of cereal by-products in human consumption.

Cereals and food grain cultivation and processing represent an important sector of the food industry [5,6,7]. Among cereals, rice, wheat, barley, and maize represent over 90% of cereal consumption [8]. However, the processing of these cereals generates significant amounts of by-products that, due to their chemical composition, deserve to be reintegrated into the circular bioeconomy system in a sustainable way (Figure 1). Barley, maize, millet, oats, rice, rye, sorghum, wheat wastes, and by-products contain a considerable amount of molecules with antioxidant properties [9] such as phenolic compounds, proteins [2,10], and bioactive peptides [11], but they also contain lipids [12], phytosterols [13], beta-glucans [14], vitamins, and minerals [15]. The recovered fractions can be used as a bioactive component in supplements, nutraceuticals, cosmetics, and pharmaceuticals, as well as food additives and other agricultural applications [16]. As a result, by-product management in the food industry can be considered a priority topic in terms of environmental protection and long-term sustainability. Even though industrial feedstock for biotechnological production of bioproducts can proficiently address the accumulation of environmental pollution caused by waste, detailed experimental trials are frequently required before scaling up the implementation due to economic factors [17]. Moreover, the bio-processing of cereal wastes into value-added products with higher functionality can reduce environmental pollution, minimize the requirement for agro-industrial waste treatment, and contribute to revenue diversification by covering multiple markets.

Overall, all the bioactive compounds recovered from cereal by-products and wastes must meet safety requirements, consumers’ acceptability, and fit into the circular bioeconomy system. In this regard, one of the most challenging decisions in isolating various bioactive components from cereal by-products is the compatibility of the matrix with extraction technologies, both from the point of view of recovery yield, purity, and the stability of the targeted compound, as well as economic feasibility and environmental impact. Other important aspects that require considerable attention are the process of reintegrating recovered compounds into functional foods, as well as ensuring the bioaccessibility and bioavailability of bioactive molecules in the human body [18,19]. Therefore, to maximize their action, improve their stability during processing and storage, protect them from unfavorable conditions, mask any unpleasant sensorial aspects, or have targeted delivery and a controlled release, different procedures can be applied before incorporation into food to solve these problems [20]. In this regard, several advanced encapsulation techniques such as spray/freeze drying, coacervation, ionic gelation, extrusion, fluidized bed coating, emulsification, and layer-by-layer deposition, have been developed and optimized in recent years [21].

As a result, this review aims to assess the recent information on the main bioactive and functional compounds recovered from cereal waste and by-products, their antioxidant, antimicrobial, and other important properties, emphasizing their functional value, applicability, and potential human health benefits.

## 2. Bioactive Compounds from Cereal Wastes and By-Products

### 2.1. Carbohydrates

Carbohydrates represent the major class of cereal constituents, being mostly composed of starch and soluble sugars but also non-digestible components (hemicellulose, cellulose, lignin, pectin, resistant starch, and other complex polysaccharides). More than 45% of the cereal bran content is made up of nonstarch polysaccharides, arabinoxylans, cellulose, fructans, beta-glucans, lignin, and its polymers, which are primarily found in the outer layer of cereals. Therefore, cereal by-products can be successfully exploited as low-cost alternatives for extracting various carbohydrates fraction with multiple potential applications for both the pharmaceutical and food industry [22,23].

The physical characteristics of dietary fibers, such as particle size, swelling and water retention capacity, water solubility, and viscosity, have a major impact on the nutritive, physiological, and technological characteristics of food, such as texture, rheology, and sensory perception [24,25]. Moreover, dietary fibers are known to have health benefits that include lowering glycemic response, controlling blood cholesterol levels, improving antioxidant activity, promoting weight loss, and enhancing the microbiota population in the small intestine and colon [26,27,28,29]. For example, arabinoxylans, recognized for their prebiotic effects on obesity [30] and other metabolic improvements [31] (e.g., the ability to lower blood cholesterol and postprandial glycemic response), represent 10.9–26.0% of the dry matter of bran [32]. The European Food Safety Authority (EFSA) acknowledged and approved in 2011 the health claim regarding the capacity of wheat arabinoxylans to reduce blood glucose levels after a meal [33].

In a recent study, Malunga and collab., suggest that antiglycemic properties of arabinoxylans extracted from wheat aleurone and bran may be derived from direct inhibition of α-glucosidase activity [34]. A similar observation was also concluded by Boll and collab., who tested the impact of bread containing a mix of arabinoxylan extracted from wheat bran and maize resistant starch, also reporting the positive influence on blood glucose response in rats and human trials [35]. It is also well known that cereal and other vegetable fiber possess a higher laxative effect compared to fiber from fruits [36]. For example, beta-glucans have the ability to prolong postprandial satiety, increased stool mass, and relieve constipation [36,37]. Similar effects were observed by Nguyen and collab., who concluded that corn bran arabinoxylans consumption had a positive effect on bowel movement and fecal consistencies [38]. Furthermore, they are recognized for their prebiotic effect and good capability to develop good colonic microbiota [39], which contribute to the production of several bioactive postbiotics, such as exopolysaccharides (EPS, e.g., dextrans, levans, fructans, and reuterans), and reduce the growth of deleterious microorganisms.

Out of the main representative cereal by-products, brewer’s spent grain (BSG) represents an important source of carbohydrates that remains unexploited in the brewing process [40,41]. With ca. 85% of the total generated by-products, BSG is mostly composed of non-starch polysaccharides (approximately 50% of dry BSG) but also contains various ratios of residual undigested starch (1.3–10%) [22]. The non-starch polysaccharides of BSG are mainly made of cellulose (15.1–25%), hemicellulose (24.8–40%), and lignin (7–28%), amounts which depend on grain cultivar and quality, adjuncts added, and conditions and efficiency of the steps involved in the brewing process. Hemicellulose, which includes xylan, glucuronoxylan, arabinoxylan, glucomannan, and xyloglucan, belongs to a group of heterogeneous polysaccharides that help the strengthening of the cell wall by interacting with cellulose and/or lignin. Commonly found in hemicellulose are glucose, xylose, mannose, galactose, rhamnose, and arabinose [42].

Regarding the presence of beta-glucans in the biomass of BSG, the values are relatively low (0.4–1.1% dry mass) due to the extensive degradation caused by processing [43]. Additionally, the malting and mashing procedures change the molar mass, molar mass distribution, and solubility of beta-glucans, partially altering their physicochemical and technical properties.

Arabinoxylans, the main structural polysaccharides of cell walls, represent the major part of hemicelluloses present in BSG (22.2% of the dry material). Due to this aspect, many studies have analyzed their impact on human health, reporting a series of potential beneficial effects such as improving cholesterol metabolism, regulating intestinal transit, inhibiting pathogenic bacteria, and reducing the postprandial glycemic response [44]. In a recent study, Lynch and collab., investigated the microbiome modulating potential of BSG with a focus on the prebiotic potential of the extracted arabinoxylans. As a result, they concluded that the tested arabinoxylans positively influenced the fecal microbiota due to bifidogenic effects [45].

As already specified in the previous examples, wheat bran is another by-product with proven beneficial effects on human health [46]. This fraction represents a sustainable source of both soluble and insoluble dietary fiber that can be converted into beta-glucans and arabinoxylans, as well as digestible carbohydrates that can be converted into starch, glucose, lactic acid, succinic acid, and/or ethanol.These complex characteristics expand its applicability as a source of valuable compounds for the food, cosmetic and pharmaceutical industries. Defatted wheat germ is also a good source of fiber, pentosans, and sugars (of which about 20% are sucrose and raffinose, respectively) [47].

Fermented wheat germ extract has been promoted to contain a range of bioactive molecules that promote positive effects in cancer prevention. Therefore, in a recent study, Kon and collab., discover that ethanolic extract of fermented wheat germ with yeast extract inhibits human ovarian cancer cell growth in a dose-dependent manner [48].

Regarding the dietary fibers found in rye, they are composed of arabinoxylans, beta-glucans, cellulose, fructan, and Klason lignin, with arabinoxylans serving as the cereal’s predominate chemical constituent. Rye has several health advantages, including a lower risk of diabetes, cardiovascular disease, and several malignancies. This by-product was shown to contain more beta-glucans and fructans (4.3–5.3%, and 6.6–7.2%, respectively) than wheat bran (2.2–2.6%, 2.8–3.7%, respectively), while the amount of cellulose (5.5–6.5%) was nearly two times lower (9.3–12.1%) [48,49]. On the other hand, the most significant source of beta-glucans is not rye, oat, and barley exhibiting higher amounts [47]. Prykhodko and collab., carried out an experimental protocol through which they proposed to highlight the influence of rye kernel base bread on gut microbiota and metabolic response. They suggested that the effect of increasing gut fermentation had a positive influence on improving both the glucose response and appetite control by maintaining satiety feelings for a long period [50].

Beta-glucans account for at least 5.5% of the dry matter and about 16.0% of the total dietary fiber in oat bran, being considered among its most important constituents. Furthermore, oat bran can have its beta-glucan content increased by 1.3 to 1.7 times over whole grain by successively dry milling and sifting the grain [51]. According to Xu and collab., oats-derived beta-glucan exhibited prebiotic activity and modulate gut microbiota increasing bifidobacteria as well as acetate and propionate productions and highlighting the potential cholesterol reduction effect [52].

As previously mentioned, the recent increased interest in consuming barley-based products comes from its high content in beta-glucans [32], which along with other phytochemicals offer antioxidant, antiproliferative, glycemic index, blood sugar, and LDL cholesterol lowering, and immune-modulating effects. Due to their increased resistance to digestion and prebiotic properties, gelation characteristics, and fermentative formation of short-chain fatty acids (SCFA) such as acetate, propionate, and butyrate, a preventive role against colorectal cancer was also observed [53,54]. Even though the use of beta-glucans in pharmacology is an interesting perspective, it still has limited applicability caused by the processing costs that accompany the extraction, purification, and standardization difficulties, therefore it requires more optimization to become sustainable.

As a first conclusion, it is well known that products like white rice grain, and in general dehulled cereals, contain fewer bioactive compounds in comparison to bran by-products due to the processing technology applied [37]. Despite that, the integration of grain by-products in food consumption represents a challenging procedure due to the generally lower sensory acceptance [39]. In order to reduce these inconveniences, the most recent advances in extraction, thermal treatments, extrusion, enzymatic treatment, and fermentation allow significant improvements both in terms of chemical and sensory properties, as well as the bioavailability of nutrients and overall quality of the final products [40,41,42,43,46,47].

Recently, many studies showed the value-added potential of cereal by-products carbohydrates. They mostly include applications in the food, feed, pharma, and packaging industries (Table 1).

### 2.2. Proteins and Amino Acids from Cereal Waste and By-Products

Proteins are one of the key components required for cell growth and repair mechanisms in our body, being essential for human nutrition. In the assessment and selection of proteins based on their quality, it is important to consider the digestibility, the amino acid profile, and the presence of antinutritive compounds besides the total protein content of raw materials [58]. Recently, food processing by-products have gained certain attention, among which, cereal-related by-products have become very attractive considering that they can be an economical source of important compounds like minerals, antioxidants (such as polyphenols and vitamins), dietary fibers, proteins, carbohydrates, and sugars [22]. As a result, many researchers present a keen interest in valorizing cereal by-products as protein sources, creating a feasible option to produce and ensure a sustainable protein source for the global demand [59]. There are several industrial-scale cereal by-products with high protein content and among these, brewer’s spent grain, rice bran, wheat bran, and corn bran proved to be excellent raw materials for producing hydrolysates with potential biological activity. Numerous studies published in the literature underline the possibility of obtaining peptides from brewer’s spent grain and rice bran protein having bioactive properties, highlighting the high-value potential protein extracted from these by-products [60,61]. Bioactive peptides are made of 2–50 amino acids that impact human health and food products [62,63]. They are classified into endogenous, and exogenous peptides (both obtained by enzymatic hydrolysis) [64]. The bioactive peptides are absorbed in the intestine and, from there, are transported through into the blood circulation.

The conversion of carbohydrates during alcoholic fermentation lowers the mass of grain leading to the increase of the amino acid content of cereal by-products which was determined to be between 30% and 85% of total protein [65,66]. In general, the proteins found in cereal by-products are classified based on their solubilities. Albumins are water soluble, globulins are soluble in salts, glutenins dissolve in alkaline solutions, and prolamins in different concentrations of alcoholic solutions. Prolamin-type proteins are present in the main cereals’ by-products (corn, wheat, sorghum, and barley by-products) and constitute around 80% of the total protein. Several research groups around the world target prolamin functionalization by extortion from cereal by-products [65].

From a biological point of view, the high essential amino acid content confers to bran proteins a higher nutritional value because these proteins play an important role during seed germination. On the other hand, cereals and cereal by-products have a variable amino acid content, depending on several factors such as plant variety or external environmental conditions [22]. In addition, it is well known that temperature rise during milling can affect the quality of proteins, but if the milling process does not generate large amounts of heat, protein denaturation can be avoided. Opposite, the proteins in brewer’s spent grains or dried distiller’s grains can be partially subjected to degradation, aggregation, or even denaturation and, in some instances, can be used as amino acids or nitrogen (N) sources. Considering the importance of these cereal-related proteins, several studies in the literature focus on the development of efficient extraction methods. Among these methods, extraction in alkaline conditions linked with isoelectric precipitation has been the most applied technique (Table 2). Due to their high protein content, the cereal’s by-products presented in Table 2 can be processed and exploited to obtain different functional ingredients/products.

### 2.3. Vitamins and Mineral Microelements from Cereal Wastes

Human and animal bodies require an optimal nutritional balance. According to existing research, human metabolism requires 49 essential nutrients to maintain health and wellbeing, including 16 mineral microelements and 13 vitamins [81,82,83]. Cereal waste and by-products are a valuable source of vitamins and microelements. The majority of micronutrients are present in bran, especially in the aleurone layer, and in cereal germs [15]. According to the requirements of the human body, the mineral elements contained in cereals and cereal by-products substrate are grouped into two categories: macrominerals (Ca, Mg, K, Na, Cl, P, and S) and oligo-minerals (I, Zn, Se, Fe, Mn, Cu, Co, Mo, F and B) [84]. The cereal by-product minerals concentration is between 6.6–9.9%. Along with the mineral content, cereal by-products have relevant amounts of B vitamins, such as niacin, pantothenic acid, biotin, thiamin, and riboflavin [15,85]. BSG, the most common cereal by-product of the beer industry, is a rich source of carbohydrates, proteins, lipids, and smaller amounts of minerals, vitamins, and phenolic compounds [86]. The main mineral elements identified in the BSG are Phosphorus (6 × 10^3^ mg·kg^−1^), Calcium (3 × 10^3^ mg·kg^−1^), Sulphur (2.9 × 10^3^ mg·kg^−1^), Magnesium (1.9 × 10^3^ mg·kg^−1^), and smaller amounts of Potassium, Iron, Sodium, and Zinc. The primary vitamin contained by BSG is Choline (1.8 × 10^3^ mg·kg^−1^), followed by reduced amounts of Niacin, Pantothenic acid, Riboflavin, and Thiamine [86]. A proper selection of the applied processes must be made to facilitate the effective recovery of bioactive compounds from the biomass for maximized utilization of the functional compounds from the BSG. The most often performed techniques attempt to convert the primary fibers of BSG, including cellulose and hemicellulose, into fermentable sugars by chemical and enzymatic hydrolysis releasing the functional compounds bound to the lignocellulosic chain [86]. Extraction methods and certain pre-treatments applied to cereal waste and by-products can significantly increase the yield of vitamins and minerals. According to Tuncel and collab., a considerable increase in B vitamins and mineral elements was observed after integrating rice bran into bread. The bread’s Zinc (Zn), iron (Fe), potassium (K), and phosphorus (P) contents progressively increased with the addition of stabilized rice bran, while the niacin content was increased by 10% [85].

Regarding the occurrence of microelements in cereal by-products and wastes resulting from the cereal processing industry, it should be mentioned that the soil mineral nutrients concentration is the crucial factor that influences the bran minerals content [85]. Moreover, the concentration of phytic acid in the waste substrate affects the bioavailability of mineral elements in cereal by-products. Phytic acid is mostly concentrated in the bran and is considered an anti-nutritional factor [15]. The large charges acquired in the gastrointestinal environment result in the formation of a stable phytate complex that inhibits the action of phytase enzymes. In some circumstances, phytic acid forms protein linkages and inhibits α-amylase activity, resulting in lower starch digestibility [87]. Processing approaches connected to increasing nutritional qualities and sensory aspects are being developed to boost the functional food value and minimize the anti-nutritional activity of cereal by-products and waste. Phytase using, soaking, germination, fermentation, boiling, extrusion, dehulling, radiation, ultrasonic waves, and storage are among the most recent approaches used to remove the phosphorus reserve in cereal by-products and waste [88].

However, different secondary metabolites, minerals, and vitamins have been extracted from cereal waste and by-products, using various approaches. These techniques might provide an alternative strategy to expand the production of bioactive compounds for use as nutraceuticals or as components in the development of functional foods in the near future.

### 2.4. Lipids from Cereal Wastes

The lipid content of cereal by-products (Table 3) is mainly associated with the specific characteristics of the biomass, such as the cultivars/varieties/species of grains, bran particle size, the oil recovery conditions, and the method of extraction, type of extraction solvent, time and temperature. In many matrices, some of the fatty acids are bound through lipid-protein or lipid-starch intermolecular bonds, and the process of acid hydrolysis is applied for releasing these lipid compounds [89,90]. Lipids are mainly found in the bran layer and wheat germ. According to Górnaś and collab., rice bran was identified as having the major lipid content yield (189 g kg^−1^ dw), followed by wheat germ (112 g kg^−1^ dw), corn bran (74 g kg^−1^ dw), oat bran (58 g kg^−1^ dw), buckwheat bran (41 g kg^−1^ dw), spelt bran (39 g kg^−1^ dw), wheat bran (33 g kg^−1^ dw) and rye bran (27 g kg^−1^ dw) [89].

Rice lipids include unsaturated fatty acids, particularly oleic acid (45%) and linoleic acid (33%), and polyunsaturated fatty acids, such as omega-3 and omega-6, with functional properties for human health [91,92]. Rice bran’s biologically active fatty acids, along with other functional compounds like tocopherols and tocotrienols, squalene, phytosterols, polyphenols, and gamma-oryzanol, are responsible for antioxidant and anti-inflammatory activities, as well as preventing cardiovascular diseases, atherosclerosis, and hyperlipidemia [91]. Because of its high lipid content, rice bran’s functional compounds and sensory qualities may be affected by the rapid process of bran oxidation. Lipases and lipoxygenase activity intensifies the lipid oxidation process, due to the accelerated activity throughout storage [90]. For that reason, certain treatments are required to avoid natural fatty acid degradation.

Wheat germ is a byproduct of the milling process, and approximately 25 million tons of wheat germ are generated globally each year [90]. Wheat germ is a valuable by-product rich in high-value nutrients, including biopeptides, polyunsaturated fatty acids (Table 3), and functional compounds such as sterols, tocopherols, tocotrienols, phenols, and carotenoids. Therefore, wheat germ oil decreases its value after 15 days due to intense enzymatic activity that causes oxidative damage to the fatty acids compounds [93]. In addition to wheat germ, a high lipid content is also found in rice germ (25%), corn germ (9–50%), and soybean germ (4–24%) [94].

By-products from the beer industry include wasted grains, hops/tubs, and yeast. Brewers’ spent grains are the most common, accounting for 85% of brewing by-products [84,95]. The majority of BSG samples are lignocellulosic materials based on whole-grain and malt kernels, which contain fibers (50–70%), starch (1–12%) proteins (15–25%), lipids (7–10%), and ash (2–5%) [84]. Triglycerides (25,300 mg/kg), free fatty acids (6710 mg/kg), and minor quantities of monoglycerides (610 mg/kg) and diglycerides (2880 mg/kg) constitute the majority of BSG lipids (Table 3). Lower amounts of steroid molecules, such as steroid hydrocarbons, steroid ketones, free sterols, sterol esters, and sterol glycosides, were also reported [96]. Special pretreatments and bioprocesses applied on BSG can increase the lipid content of this substrate. Patel and colleagues pretreated BSG with microwave-assisted alkaline and organosolv pretreatments, followed by *Rhodosporidium toruloides* yeast fermentation for the production of microbial lipids. Following the pretreatments and the fermentation bioprocess applied, a profile of fatty acids similar to common vegetable oils was obtained. This integrated technique may be utilized to obtain biodiesel raw materials based on BSG [95]. Moreover, BSG bioactive lipids might be seen as a valuable source of fatty acids, triglycerides, and phytosterols, considering that these compounds have a wide range of functional properties and are of interest to the industrial sectors, such as nutraceuticals, pharmaceuticals, cosmetics, food, and food supplements [12,13,97]. Table 3 shows the majority of the lipid content in a variety of by-products and cereal waste.

**Table 3:** Fatty acid composition of cereal by-products and waste.

Cereal By-Product/Waste	Fatty Acid	Concentration (% of Total Lipids)	Extraction Methods	Functional Properties	Reference
Rice bran	Triacylglycerol	60.12	Solvent extraction (n-hexane)	Balanced fatty acid profile; Delicate flavor; High smoke point; High bioactive ingredient.	[92,98]
	Polyunsaturated fatty acids	40.73			
	Linoleic acid	38.84			
	Oleic acid	34.31			
	Palmitic acid	19.87			
	Free fatty acids	29.69			
	Diacylglycerol	9.98			
	Monoacylglycerol	0.21			
	γ-oryzanol	18.53			
	Phytosterol	22.40			
Wheat germ	Linoleic acid	57	Solvent extraction (hexane)	Food ingredients with potential health benefits.	[93]
	Palmitic acid	17.5			
	Oleic acid	15			
	Linolenic acid	6			
	Total polyunsaturated fatty acids	64.5–63.7			
Brewer’s spent grain	Free fatty acids	18	Soxhlet acetone extraction; Hot water extraction; Sulfuric acid hydrolysis; Alkali extraction	Nutraceutical, pharmaceutical, and cosmetic properties.	[96]
	Triglycerides	67			
	Monoglycerides	1.7			
	Diglycerides	7.7			
	Steroid compounds	5			
Oat bran	Oleic acid	44.09–46.68	Subcritical butane extraction	Preventive effects on cardiovascular disease and development of atherosclerosis; Reducing body fat.	[99]
	Linoleic acid	32.54–32.88			
	Stearic acid	1.71–1.89			
	Palmitic acid	15.68–16.03			
Corn germ	Palmitic acid	11.57	Pressing extraction	Commercial shortening replacement in food industries.	[100]
	Stearic acid	2.89			
	Oleic acid	29.45			
	Linoleic acid	54.31			
Rye bran	Linoleic acid	61.09	Supercritical carbon dioxide extraction using response surface methodology	Food grade ingredient.	[101]
	Palmitic acid	13.74			
	Oleic acid	13.65			
	Linolenic acid	6.37			
Corn waste	Palmitic acid	23.0	Solvent extraction analyzed by gas chromatography (Folch method)	Feed or pharmaceutical industry.	[102]
	Stearic acid	3.4			
	Oleic acid	11.7			
	Linoleic acid	52.9			
	α-Linolenic acid	5.3			

## 3. Compounds with Antioxidant Properties from Cereal By-Products

Cereal waste and by-products recovered compounds are widely used in the food industry (additives for prolonging food products’ shelf life) [63,103,104], cosmetics [105,106], phytopharmaceutical and health products (food supplements, nutraceuticals, adjuvants in therapies) [64,107] mainly due to their biological activities.

An antioxidant is a chemical that can slow or stop oxidation or oxidative cell damage caused by oxidants [8]. Antioxidants can disrupt the oxidation chain by stabilizing themselves via chemical structure resonance. Molecules can act as antioxidants by interacting with transcription factors [108]. Human’s most common diseases, such as cancer, diabetes, and cardiovascular or neurological problems, have all been linked to oxidative stress [107]. Moreover, food degradation and lipid oxidation have also been attributed to oxidative stress [104], all being improved by the presence of antioxidant compounds. Natural antioxidants are primarily found in vegetal sources [109]. Studies revealed that antioxidants, such as terpenes, phenols, phytosterols, and bioactive peptides, from cereal wastes, may be extracted by different methods and may be further utilized in various applications as shown in Table 4. Furthermore, cereal by-products are reported to be used as a substrate for bacteria or by fungi able or engineered to produce antioxidants [110].

As can be seen in Table 4, most of the cereal waste antioxidants refer to phenolic compounds. Proteins and peptides can also have antioxidant activities, adding them to food matrices delaying the process of lipid oxidation [123]. According to Stefanello and collab., the samples defatting negatively influence the phenolic content and the antioxidant activity (except for the corn silage). They used microwave-assisted extraction methods to determine the total phenolic content of brewer’s spent grain, corn silage, rice, corn, and wheat brans, using three different solvents (acetone, methanol, and aqueous NaOH 0.75% *v*/*v*) [124]. Another study found that using lactic acid and specific amino acids could have a significant impact on total polyphenols and antioxidant activity yield [125]. Although studies regarding the bioactive peptides isolated from cereal wastes are scattered, most experimental protocols also highlight their antioxidant potential. Thus, several other activities of bioactive peptides are being tested. Ilhan-Ayisig and his colleagues proved a cytotoxic effect on the MDA-MB-231 estrogen-independent breast cancer cell line when treated with bioactive peptides derived from rice husk. Notable is that the nonencapsulated form of the peptides has proven anticancer potential (IC_50_ values of >100 μg/mL for all formulations in normal cell line VERO) [11]. In another study, non-alcoholic steatohepatitis was ameliorated in mice by upregulating the AMPK/ACC signaling pathway. The mice were given leucine-arginine-proline and leucine-glutamine-proline (from wheat bran) in water solutions (0.05 and 0.20%) for 10 weeks [120]. These leucines may impact the modulation of insulin sensibility [126].

Over the antioxidant activity, the cereal waste recovered compounds also present anticancer, antidiabetic, anti-inflammatory, or antimicrobial functions [109,114,115,116]. Wheat bran oil proved antibacterial activities against pathogenic bacteria such as *Escherichia coli*, *Pseudomonasaeruginosa*, *Bacillus subtilis,* and *Staphylococcus aureus* and some fungi such as *Candida albicans* and *Aspergillus niger* [127]. Furthermore, free phenolics extracted from wheat bran using ultrasound-assisted extraction, as well as bound phenolics extracted using alkaline hydrolysis and ultrasound-assisted alkaline hydrolysis, expressed antimicrobial activity against *Staphylococcus aureus* and *Staphylococcus epidermidis* strains [115]. BSG extracts integrated into biofilms showed antimicrobial activity against Gram-positive (*Staphylococcus aureus*) and Gram-negative (*Escherichia coli*) bacteria, as well as antifungal efficacy against the polymorphic fungus *Candida albicans* [128]. 

Anti-inflammatory plant-derived bioactive chemicals are also in high demand. Immunomodulatory substances impact the immune system’s reaction to various distressing factors, either favorably or unfavorably. Inflammation is at the root of many medical illnesses, including Parkinson’s disease, Alzheimer’s disease, dementia, multiple sclerosis, and autoimmune diseases [129,130]. The anti-inflammatory activity of eight phenolic compounds isolated from BSG (pale and black) was investigated. The results demonstrated that phenolic extracts of BSG, particularly pale BSG extracts, can inhibit the stimulated production of cytokines (particularly interleukin-2, interleukin-4, and interleukin-10) and protect against cellular oxidative stress [130]. In addition, McCarthy and colleagues successfully proved the antioxidant and anti-inflammatory action of protein hydrolysates derived from BSG [131]. Furthermore, wheat bran ingestion lowered the inflammatory effect on the liver. The size of the ingested particles impacted the lowering of hepatic and systemic inflammatory indicators following high sugar (fructose) consumption, varying the activity of the inflammatory intestinal barriers.

As a result, cereal by-products can be considered sustainable sources of bioactive compounds and secondary metabolites with multiple functionalities, therefore their incorporation into functional foods, supplements, and pharmaceutical products is a research field that requires increased attention.

## 4. Conclusions

Cereal waste and by-products generated during cereal processing include bran, husk, germ, hops, hulls, and brewer’s spent grain components, which can be collected and valorized under a circular economy strategy. These by-products contain a variety of high-value compounds, mainly bioactive compounds with significant health benefits. They can be exploited as food ingredients, supplements, additives, or extracts that are high in functional molecules and micronutrients, such as phenolic compounds, novel carbohydrates, carotenoids, biopeptides, bioactive fatty acids, amino acids, prebiotics, vitamins, and mineral elements. Bioactive compounds derived from cereal waste and by-products can be used as antioxidants and preservatives, reducing lipid oxidation and microbial growth. As a result, bioactive compounds derived from cereal by-products can increase the product shelf-life, with potential applications in the food additives, cosmetic and pharmacology industries, animal products, dairy products, beverages, and bakery products industries. Furthermore, processing technologies designed to improve nutritional characteristics and sensory features are being developed to increase the functional food value, and nutrients bioavailability, while reducing the anti-nutritional factors of cereal by-products and waste. In the near future, more studies are necessary on the extraction procedures in order to provide a sustainable strategy for increasing the production of bioactive compounds for use as nutraceuticals or as ingredients in the development of functional products.

## Figures and Tables

**Table 1:** The main carbohydrates composition of different grain by-products and their potential application.

Compounds	By-Product	Concentration	Industrial Applications	Health Benefits	References
Residual undigested starch	BSG	1.3–10%	Production of fungal biomass and ethanol; Development of prebiotic ingredients for the meat industry	Positive effects on metabolism regulate the fermentative processes in the colon and increase the levels of glucagon-like peptide-1, known for its anti-diabetic and anti-obesogenic features	[22,26,27,28]
Beta-glucans	Oat bran	5.5% dry matter	Using supercritical carbon dioxide to remove the oat bran lipids can increase by more than 40% the beta-glucan level; Incorporated high molecular weight oat beta-glucan into milk to obtain calorie-reduced and cholesterol-lowering dairy products; Increase of beverage satiety capacity; Ingredient for wheat flour substitutes; Food hydrocolloids; Wound dressing products; Curing partial-thickness burns; A bone-substituting material; Novel prebiotics; Film-forming moisturizer; Skin and dermatological compositions; Cosmetic product; Animal and fish feed additives	Antioxidant and antiproliferative activities, regulate the glycemic index and blood sugar and reduce LDL cholesterol. Immune-modulating effects, prophylactic roles against colorectal cancer, prolong satiety and have prebiotic effects, facilitating the elimination of fecal matter and avoiding constipation problems Anti-inflammatory, skin-care effects	[22,28,29,47]
	BSG	0.36% dry matter			
Arabinoxylans	Different cereals bran	10.9–26.0% of the bran dry matter	Food-thickening and stabilizing agents and films for the food industry (packaging materials); Controlled release of bioactive compounds.	Prebiotic effect, reduce the risk of metabolic disorders such as obesity, have the ability to regulate the postprandial glycemic response and stabilize cholesterol levels Minimizes the risk of developing diabetes and chronic heart disease Anticarcinogenic properties	[30,31,33,47]
Cellulose	Rye bran	5.5–6.5%	Feed supplement Paper packaging containers	It facilitates the shortening of the intestinal transit time and also the elimination of possible carcinogens, which contributes to reducing the risk of developing colon cancer.	[22,47,53,55,56]
	Wheat bran	9.3–12.1%			
	BSG	15.1–25%			
Lignin	BSG	7–28%	Food industry (dispersing, binding, and emulsifying agent), food supplement, animal feed and medicine, construction industry, cosmetic products, crop protection (lignin-based pesticides), printing ink	Anticarcinogenic, antimicrobial, and antioxidant properties, increase fecal bulk and stimulates intestinal transit, can undergo fermentation when exposed to colon microbiota, anti-hyperlipidemia and anti-obesogenic agent, protective activity against oxidative stress and inhibition of LDL oxidation	[22,33,47,53,57]
	Wheat bran	3.3–4.9%			
	Corn bran	10 g/kg			

BSG—brewers’ spent grain.

**Table 2:** Bioactive proteins and amino acids recovered from cereal by-products and waste.

Cereal Waste	Protein/Amino Acids Quantity	Extraction Methods/Treatments	Extraction Efficiency/Yield	Properties/Applications/Other Observations	References
Brewers’ Spent Grain (BSG)	Protein: 23.10 g/100 g dw for pale BSG Protein: 26.93 g/100 g dw for black BSG	Sequential aqueous and alkaline (110 mM NaOH) extraction, followed by isoelectric precipitation (pH 3.8)	Pale BSG: 59% protein extraction yield Black BSG: 15% protein extraction yield	Protein-enriched isolates can be used as bioactive ingredients for incorporation into conventional and functional foods.	[67]
	Protein: 23.4 g/100 g BSG dw	Enzymatic (Alcalase 2.4 L) and ultrasound-assisted enzymatic extraction (amplitude 40%, treatment time 10 min, pulse 5 s:3 s off)	61.6% recovery for enzymatic treatments and 69.8% recovery for ultrasound enzymatic extraction	Ultrasound pretreatment increases the efficiency of protein separation, reduces enzyme loading, and decreases enzyme incubation time.	[68]
	Protein: 22.63 g/100 g defatted BSG	Acid pretreatment (one-step dilute acid pretreatment with the acid solution (11,400 mg H_2_SO_4_/g BSG) autoclaved at 121 °C for 1 h) Hydrothermal pretreatment (a. 60 °C/24 h, shaker incubator—250 rpm)/(b. 25 °C, 1.5 h)	Protein extraction efficiency 90% Protein extraction efficiency: 64–66% (a) and 43% (b)	Even though the acid treatment had a higher efficiency, a significant amount of carbohydrates and lignin was also solubilized together with protein; instead, the hydrothermal pretreatment had a better selectivity and is more environmentally friendly.	[69]
	Protein: 22.9 g/100 g defatted BSG	Sodium hydroxide treatment 5% (*w*/*w*) Alcalase treatment (20 μL/g dry BSG) Sodium bisulfite treatment (5% *w*/*w*)	Protein separation efficiency 81.8% Protein separation efficiency 83.7% Protein separation efficiency 68%	Enzymatic treatment proved to be the most effective and the resulting protein concentrate had also the highest lysine content (4.1%, *w*/*w*).	[70]
	Protein: 24.70 g/100 g dw	Sodium hydroxide (110 mM) and ultrasound treatment (power 250 W, duty cycle 60%, 20 min/25 °C)	Extraction yield of 86.16% and purity at 57.84%	Plant-based protein source to the food industry. Improved fat absorption capacity, emulsifying, and foaming properties.	[71]
	Amino acids: 43.62 mg/g^−1^ proteins	Subcritical water hydrolysis in a single reactor (120 min at 15 MPa, 5 mL water min, 80–180 °C, solid: fluid of 20 g^−1^ BSG)	The main amino acids of hydrolysate: tryptophan 215.55 µg mL^−1^, aspartic acid 123.35 µg mL^−1^, valine 64.35 µg mL^−1^, lysine 16.55 µg mL^−1^, and glycine 16.1 µg mL^−1^	Applicability in the field of food and supplements production	[72]
Rice bran defatted (RBD)	Soluble proteins: 8.23 g/100 RBD	Alkaline extraction of proteins and fractionation by the Osborne method	55.8% of the total soluble proteins, of which 6.1%albumin, 4.5% globulin, and 43.5% glutelin.	Applicability in the field of food and supplements and cosmetics production.	[73]
	Protein: 15.67 g/100 g RBD	Alkaline extraction (60 min, pH 11, 55 °C) Microwave-assisted extraction (120 s; pH 11, 55 °C)	Protein content of concentrated product 75.32% and extraction yield 12.85% Protein content of concentrated product 79.98% and extraction yield 15.68%	Comparing the two methods, the microwave-assisted one proved to be more efficient and environmentally friendly. Also, the microwaves did not affect the extracted rice bran proteins.	[74]
	Protein: 14.13% of concentrate product	Microwave-assisted extraction (1000 W of MW power, extraction time 90 s, solid to liquid ratio of 0.89 g rice bran/10 mL of distilled water) and response surface methodology	Protein content of concentrated product 71.27% and recovery yield 22.07%	Food industry—strong antioxidant activity. MAE is considered an environmentally friendly technique.	[75]
Malted barley germs (MBG)	Protein: 29.1% on a dry matter basis	Amino acid profile by LC/fluorescence	Total amino acid 214 mg/g of which 35–40% are essential (leucine 15.7 mg/g, valine 13.5 mg/g, lysine 11.7 mg/g, and arginine 12.5 mg/g dw	Valuable source of good quality nitrogen fraction. Applicability in the field of food and supplements production.	[76]
Brewing cake	Protein: 30.4% on a dry matter basis	Amino acid profile by LC/fluorescence	Total amino acid content 238 mg/g of witch 35–40% are essential amino acids (leucine 18.0 mg/g), phenylalanine 14.6 mg/g, valine 13.3 mg/g, cysteine 11.3 mg/g, arginine 12.1 mg/g dw	Valuable source of good quality nitrogen fraction. Applicability in the field of food and supplements production.	[76]
Wheat bran (WB)	Protein: 17.2 g/100 g WB dw Total amino acids (AA): 12.5 g/100 g WB proteinTotal essential amino acids (EAA): of 4.28 g/100 g WB protein	Alkaline extraction (pH 9.5, 2 h, followed by isoelectric precipitation, pH 4.2)	Wheat bran concentrate (WBPC) protein content: 61% Protein recovery yield: 20.5–24.8% Total AA of WBPC 60.11 g/100 g), total EAA 22.79 g/100 g	WBPC showed excellent functional properties in terms of high solubility, good water, and fat absorption capacity. Balanced amino acid composition, high in essential amino acids, with good levels of lysine and threonine, and phenolic acids.	[77]
Defatted Wheat Germ (DWG)	Protein: 34.9% dw (albumin 34.5% globulin 15.6%, glutelin 10.6%, and prolamine 4.6%)	Alcaline extraction (pH 9.5 with 1 M NaOH, stirring 30 min, the supernatant was adjusted to pH 4.0 with 1.0 M HCl to precipitate the proteins, washed and adjusted to pH 7.0 using 0.1 M NaOH, then freeze-dried)	Isolate protein content 88.5%, recovery yield in the range of 24.0–37.0%	Significant level of essential amino acids. DWG can be considered a good vegetable protein supplement for cereal-based diets.	[78]
Defatted corn germ (DCG)	Protein: 12.48% fresh weight basis	Alkaline extraction of corn germ partially defatted by supercritical fluid extraction	Protein content of DCG concentrate 48.5% dry base reported Yield of protein extraction 21.3%	Good foaming capacity and stability	[79]
Defatted oat bran (DOB)	Protein: 17.6%	Enzyme-assisted extraction (Viscozyme L, pH 4.6, incubation time 2.8 h, and temperature 44 °C)	Extraction yield 56.2%	Applicability in the field of food and supplements production.	[80]

dw—dry weight basis; fw—fresh weight basis.

**Table 4:** Antioxidant compounds recovered from cereals wastes and by-products.

Cereal Waste	Antioxidant Compounds	Extraction Methods/Biotechnology	Extraction/ Production Yield	Antioxidant Activity	Application	References
Corn silage	Polyphenols	Enzymatic treatment	412.83 mg GAE/100 g	2961.6 μM (ABTS)	-	[111]
Brewers’ spent grain	Phenolic compounds	Supercritical carbon dioxide	3 g mass of extract	2% DPPH		[112]
	Polyphenols	Acidifies solution (pH 2,5–3)	1.14 mg GAE/g	8–13%	-	[113]
Oat bran	Protein hydrolysates	Hydrolyzed with Flavourzyme (1), Papain (2), or Alcalase (3)	89–93%	627.17 (1); 682.90 (2); 652.67 (3) µM TE/g (ORAC)	-	[114]
Rice bran	Free phenols Bound phenols	Ultrasound-assisted extraction (65% ethanolic solution) Ultrasound-assisted alkaline hydrolysis	17–20%	275.1 (DPPH) IC_50_ (μg/mL) 38.01 (DPPH) IC_50_ (μg/mL)	Cosmetic formulation	[115]
	Protein hydrolysates	Hydrolysate by Alcalase 2.4 L and Protease 500 G	79.12%	75–90% (DPPH)	-	[116]
	Protein hydrolysates	Protein enzyme-assisted extraction/hydrolysis	-	2.8 μmol TE/g (DPPH)	Food additive	[117]
	Polyphenols	Glycerol extraction	708.58 ± 12.36 mg GAE/100 g dw	700.35 mg GAE/100 g	-	[118]
Sesame bran	Phenols	Microwave-assisted enzymatic extraction	-	1.94 µmol TE/g	Functional food ingredient	[119]
Wheat bran	Free phenols Bound phenols	Ultrasound-assisted extraction (65% ethanolic solution) Ultrasound-assisted alkaline hydrolysis	17–20%	1194.8 (DPPH) IC_50_ (μg/mL) 3.61 (DPPH) IC_50_ (μg/mL)	Cosmetic formulation	[115]
	Peptides	HPLC purification	-	3000–3300 μmol/L biological antioxidant potential (free radical analyzer system)	Antidiabetic compound	[120]
Wheat and rye waste (distillery stillage)	Polyphenols	Conventional solid-liquid extraction (1) Ultrasound-assisted extraction (2) Microwave-assisted extraction (3)	52–99%	10.84 (1); 16.67 (2); 26.73 (3) μmol TE/g (ABTS) 10.84 (1); 2.95 (2); 5.57 (3) μmol TE/g (DPPH) 36.73 (1); 5.57 (2); 3.71 (3) μmol FeSO_4_/g (FRAP)	-	[121]
Wheat waste	Astaxanthin	Solid state fermentation	17–109%	90–95% of the antioxidant (DPPH) activity of astaxanthin from plant	-	[110]
Wheat and Oat Bran	Phenolic compounds	Ultrasound-assisted extraction	25–50 mg GAE/100 g	40–52% (DPPH)	-	[122]

“DPPH”—2,2-diphenyl-1-picrylhydrazyl; “IC_50_”—half maximal inhibitory concentration; “-“ not mentioned; “TE”/“GAE”—trolox/gallic acid equivalent; “ORAC”—oxygen radical absorbance capacity; “ABTS”—2,2′-azino-bis(3-ethylbenzothiazoline-6-sulfonic acid); “FRAP”—ferric reducing antioxidant power assay.
